# Factors Contributing to Employment Status over Time for Caregivers of Young People with Mental Health Disorders

**DOI:** 10.3390/healthcare10081562

**Published:** 2022-08-18

**Authors:** Ana María Brannan, Eileen M. Brennan, Claudia Sellmaier, Julie M. Rosenzweig

**Affiliations:** 1 School of Education, Indiana University, Bloomington, IN 47405, USA; 2 School of Social Work, Portland State University, Portland, OR 97201, USA; 3 School of Social Work and Criminal Justice, University of Washington, Tacoma, WA 98402, USA

**Keywords:** parental employment support, work–life balance, child mental health services, caregiver strain

## Abstract

This study utilized the conservation of resources theory to guide the examination of employment outcomes for caregivers of children with emotional and/or behavioral disorders. The sample included 2455 caregivers whose children received services through federally funded systems of care. Of special interest was whether receiving services and supports predicted change in employment status. We examined change in employment between baseline data collection and the six-month follow-up including: (1) gaining employment, and (2) retaining employment. Findings indicated that the relationship between service/supports and caregiver employment differed depending on initial employment status, and type of service received. Accessing any service was associated with gaining employment. For families who accessed any services, receiving behavioral aide services was associated with gaining employment. Caregivers of children who used residential services were less likely to lose employment. Several child, caregiver, and demographic variables also predicted employment status over time. Taken together, the findings suggest that caregivers of children with emotional and behavioral challenges are at risk for downward cycles of resource loss, and that services and supports have the potential to mitigate that risk.

## 1. Introduction

The conservation of resources theory holds that individuals seek to acquire and protect resources [1]. Resources can include tangible assets (e.g., money, income, secure well-paying jobs), but they can also be intangible (e.g., psychological well-being, good physical health, social support). When resources are threatened, stress results, which leads individuals and families to utilize current resources to respond to, and prevent, further resource loss. Resources tend to aggregate, existing not in isolation but in “caravans [1]” (p. 312). Those with greater resources are better protected; they are better able to reduce risk of loss, as well as use existing resources to garner more resources. Conversely, those with fewer resources are at greater risk of resource loss because they have fewer current resources to protect them in the face of further loss. Increasing depletion of resources leads to greater stress, which can lead to heightened cycles of resource loss over time [2]. In addition, research suggests that acute resource loss can impact psychological well-being more profoundly than more stable and chronic lack of resources [3,4]. The conservation of resources theory holds that while acquisition of additional resources only modestly affects well-being in the face of stressful situations, resource gains are particularly protective against stress in the face of resource loss [1].

Applied to families of children with mental health challenges, loss or reduced parental employment is one way that resources are reduced, including those needed to care for the child, access services and supports, and meet other family needs [5]. Alternatively, gaining employment may serve to bolster the family’s ability to respond effectively to the demands of caregiving, prevent further resource loss, and protect against stress. In this study, we apply the conservation of resources theory to examine factors associated with employment outcomes over time among caregivers of children with emotional and behavioral disorders.

Having a child with emotional and behavioral disorders threatens the acquisition and maintenance of resources to the extent that it requires families to provide *exceptional care*, including obtaining specialized services, and committing an inordinate amount of time and energy to caregiving [6,7]. According to the conservation of resources theory [1], families raising a child or youth with emotional and behavioral challenges who have more resources (e.g., more income, more flexible jobs, better work benefits, more extensive community services) are able to apply them to protect the family from further resource loss [8]. For example, families with greater resources may have better health insurance to pay for specialized services. They also may have more flexible schedules so that caregivers can respond to problems at school or day care with less work disruption [9]. Families with more resources may also be able to hire a nanny with specialized skills when their child’s behavioral challenges prevent them from utilizing traditional childcare services. Families with fewer resources, however, are at risk of falling into an accelerating cycle of loss because they are less able to use their meager assets to protect themselves from further resource loss associated with exceptional caregiving.

One way that exceptional caregiving can threaten a family’s resources is through its impact on employment. Raising a child with emotional and behavioral disorders potentially reduces a caregiver’s ability to participate in the paid labor force [10,11]. For example, caregivers may need to miss work to deal with problems at school or to take the child to treatment. Often, excessive caregiving demands conflict with work responsibilities, leading caregivers to reduce work hours or leave their jobs [12,13,14]. In their survey of employed caregivers of children with mental health challenges, Rosenzweig and Huffstutter [15] found that many had quit a job (48%) or been terminated due to their care responsibilities (27%). Another study found that, compared to working parents, parents who were not employed were caring for children who missed more school days and had less access to adequate child care because of the severity of their symptoms [10]. 

Exceptional caregiving affects employment outcomes for both men and women; however, the negative impact is greater for women. Among employed caregivers of adults with disabilities, women are more likely than men to quit their jobs, reduce work involuntarily, and retire early [16,17]. Women also report more wage and income loss as a result of exceptional caregiving demands [17]. Research suggests that these differences are related, in part, to family and societal expectations regarding gender roles [13]. 

In addition to its impact on employment, caring for young people with serious mental health challenges can have other negative effects. These additional hardships have been termed *caregiver strain* [18]. In addition to global caregiver strain, three related but distinct types of caregiver strain have been identified including: *objective* (e.g., financial demands, disrupted family relationships), *subjective internalized* (e.g., feeling exhausted, sad, guilty), and *subjective externalized* (e.g., feeling anger, embarrassment, resentment). Greater child symptom severity is associated with heightened caregiver strain [19,20,21,22]. Some demographic variables are related to caregiver strain. Most notably, African American caregivers have consistently been found to report less strain than caregivers of other racial backgrounds [23,24]. Findings are mixed for other demographic variables. Caregivers of boys and older children have been found to report greater strain [21,25], but not in all studies [26]. In most studies, broad markers for resources, such as income and socioeconomic status, have not consistently been found to be significant predictors of strain [21,25]. Caregiver education has been found to be positively related to caregiver strain in some studies [27], but not others [21,28].

However, when resources are defined more specifically, relationships have been found between caregiver strain and availability of tangible and intangible resources [29]. Greater caregiver strain is associated with families not being able to meet basic needs (e.g., access to two meals a day, money to buy necessities, enough clothes). Caregivers who reported greater strain also reported having less adequate housing, utilities (e.g., electricity, water), child care, and benefits. Caregiver strain was also found to be associated with less time for meeting social needs (for example, time to socialize), and for self-care (e.g., time for exercise). It is notable that these findings were consistent across economically diverse families with significantly different levels of income [29]. 

Research also suggests a link between caregiver strain and caregiver employment. In a study examining the predictors of caregiver strain, Sales and colleagues [28] found that working more hours was negatively related to global caregiver strain. Using employment outcomes as dependent variables, others have found greater objective and subjective internalized strain to be associated with less workforce engagement [22]. However, subjective externalized strain appears to operate differently than the other two dimensions. Caregivers who experienced greater subjective externalized strain reported being employed for more months, working more hours per week, and missing fewer days of work due to the child’s challenges [22].

Families of children with emotional and behavioral disorders often utilize formal and informal services to support their child and protect against resource loss for the family. However, few studies have examined the extent to which mental health services and related supports impact caregivers’ ability to participate in the paid labor force. In one preliminary study, the majority of caregivers reported that several types of mental health services received through federally funded systems of care (e.g., including medication management, family therapy, and family support) improved their “ability to do their job” [30]. A later study from the same system of care initiative found that in the six months following entry into systems of care, average days missed from work due to the child’s challenges dropped from 4.8 to 3.5 per week [31]. A more recent study found that the patient-centered medical home model of pediatric care reduced risk for employment problems among caregivers of children with attention-deficit hyperactivity disorder [32].

Taken together, the body of evidence suggests that families caring for children with mental health challenges are at risk for downward cycles of resource loss. The negative relationship between caregiver strain and resources is potentially exacerbated by the risk of poor employment outcomes. The research suggests that services and supports may have the potential to reduce caregiver strain and improve employment engagement, thereby mitigating the risk for accelerating cycles of resource loss.

In this study, we applied the conservation of resources theory [1] to examine what family resources are related to change over time in employment among parents of children with emotional and behavioral disorders. Receipt of formal and informal services is a key resource of interest in this study. Specifically, we examined whether services led to better employment outcomes, and explored what other resources contributed to work engagement. The employment outcomes of interest included whether caregivers gained or retained employment.

## 2. Materials and Methods

This study conducted secondary analysis on data collected as part of a longitudinal outcome study of the national evaluation of the Comprehensive Community Mental Health Services for Children and their Families Program [33]. The Substance Abuse and Mental Health Services Administration (SAMHSA) funded this program to promote the implementation of systems of care for children with emotional and behavioral disorders. Data for this study were collected during Phase V of the national evaluation between the years of 2004 and 2011. Families were recruited into the study within 30 days of their child’s intake into mental health services. This is a convenience sample and data are not weighted to represent the population of children receiving mental health services.

This study used data collected at baseline (i.e., within 30 days of the child entering services) and at 6-month follow-up. At baseline and follow-up, caregivers reported employment for the previous six months. Hence, the observation period ranged over 12 months (from 6 months before baseline to 6 months after baseline). Fifty-six communities participated in Phase V of the national evaluation. However, some communities struggled to implement the evaluation as designed. Some reasons for these challenges included: serving more transient families; working in rural and frontier communities; and lack of structural resources (e.g., no local college or other partner with evaluation experience). Because of this, we only used data from the 18 communities that had retention rates of 75% or higher between baseline and follow-up data collection.

### 2.1. Participants

Families included in this study were enrolled in the longitudinal outcome study (i.e., operationalized as having completed the baseline child clinical severity assessment). Of the 3291 families with baseline clinical severity data, 2455 (74.6%) families had complete data on all measures used in these analyses. Using the available data, we found some differences between families included in analyses and those excluded. Compared to caregivers excluded from analyses, those included were more likely to be female (χ^2^ = 8.49 (1), *p* < 0.01), and reported greater objective strain (*t* (2597) = −2.03, *p* < 0.05), subjective externalized strain (*t* (2597) = −2.02, *p* < 0.05), and subjective internalized strain (*t* (2597) = −2.87, *p* < 0.05). Children included in analyses had more severe internalizing symptoms (*t* (3289) = −1.98, *p* < 0.05), and more severe externalizing behavior (*t* (3289) = −2.41, *p* < 0.05). No differences between included and excluded families were found for caregiver education, race/ethnicity, number of children in the household, or family income.

Most caregivers in the included sample were women (93.2%). The majority of caregivers were biological parents (80.5%); most other caregivers (17.5%) were adoptive or step-parents, or other relatives (e.g., siblings, grandparents, aunts). Caregivers’ ages ranged from 18 through 80, with an average of 40 years old (*SD* = 10.00). Sixty-five percent were White, 29% were African American, and 6% were of some other race or were of mixed race. Seventeen percent of caregivers were Hispanic/Latino (of any race). Most of the children in the sample were boys (64.6%) and ranged in age from 5 to 20 (mean age = 11.76, *SD* = 3.23). Sixty-five percent of the families had annual household incomes of less than USD 25,000; thirty percent had annual incomes less than USD 10,000. 

### 2.2. Measures

All data used in this study were collected from caregiver respondents. Data were collected in face-to-face interviews. Caregivers were given choices of location for the interview that were convenient for the respondent but provided sufficient privacy. These included the family’s home, the child’s school, convenient local venues (e.g., libraries), and the evaluation office.

#### 2.2.1. Employment Outcome Variables

Employment status over a 12-month period was the outcome variable of interest in this study (i.e., from 6 months before baseline until 6-month follow-up data collection). We constructed two employment outcome variables from one item collected at baseline and at six-month follow-up (i.e., “At any time in the past 6 months, did you have a paid job, including self-employment?”). The first outcome variable captures having gained employment. Caregivers who reported not working in the six months before baseline, but then reported at follow-up that they had worked in the previous six months (i.e., since entering the study), were identified as having gained employment (i.e., 1 = gained employment, 0 = did not gain employment). The second outcome variable was retained employment. Caregivers were identified as having retained employment if they reported having worked in the six months prior to baseline and reported working in the six months before the six-month follow-up data collection point (i.e., 1 = retained employment, 0 = lost employment). 

#### 2.2.2. Predictor Variables

Variables used to predict employment status over time included: service use by child and/or family; child clinical severity; caregiver strain; and child, caregiver, and family demographic variables. These are described below.

*Service use data.* Data on service use were collected at six-month follow-up and questions asked caregivers about services received in the previous six months. In the first set of analyses, we examined whether use of any service by the child and/or family was related to employment outcome (i.e., use of any service = 1, no use of service = 0). The second set of analyses considered what types of services were associated with caregiver employment outcomes. Services used in these analyses included: (a) behavioral aide; (b) psychotropic medication; (c) day treatment; (d) residential services (i.e., inpatient hospitalization, residential treatment facility, therapeutic foster care, therapeutic group home); (e) formal family services (i.e., family therapy, family preservation services); and (f) family support services (i.e., respite, flexible funds for non-traditional support, transportation, peer-to-peer support). Each of the six types of services was coded separately (i.e., 1 = use of the specific service, 0 = no use of the specific service). All families were included in models examining use of any service. Only families who received any service were included in analyses on types of service used. We did not examine the impact of receiving clinical evaluation, traditional outpatient counseling, or case management, as those were used by virtually all families who received any service.

*Child predictor variables.* Child characteristics included as predictors were clinical severity and child age. Severity of internalizing and externalizing symptoms was assessed using the Child Behavior Checklist (CBCL) [34]. The CBCL has been used extensively in mental health services research and has demonstrated good validity and reliability [34]. This study used the two broad band scale T scores of the CBCL, internalizing symptoms subscale (e.g., depression, worry, fear, anxiety), and externalizing symptom subscale (e.g., conduct-related problems, hyperactivity, aggression). The CBCL gathers the caregiver’s report of 112 child symptoms on a 3-point scale: 0 (not true), 1 (somewhat or sometimes true), or 2 (very true or often true). Higher scores indicate greater symptom severity. The clinical cut-off for the CBCL is 64 for internalizing and externalizing symptoms. Because child age has been found to be related to caregiver employment [35,36], child age was included in analyses as a control variable. 

*Caregiver predictor variables.* Caregiver strain and demographic variables were used as predictors in the analysis. We used the Caregiver Strain Questionnaire (CGSQ) [18] to assess caregiver strain. The CGSQ contains 21 items that assess strain experienced by caregivers of children with emotional and behavioral disorders. Caregiving difficulties that result from their child’s challenges are rated by caregivers using a 5-point scale ranging from 1 (not at all a problem) to 5 (very much a problem). The CGSQ assesses strain along three dimensions (i.e., subscales) including: objective strain (i.e., observable disruptions and onerous events), subjective internalized strain (i.e., internalized feelings of sadness, worry, fatigue), and subjective externalized strain (i.e., externalized feelings of anger, resentment, embarrassment). The CGSQ has been widely used in research studies and has demonstrated good reliability and validity with diverse populations [18,23,26,28,37].

Several caregiver demographic characteristics found to be related to employment were included in the analyses as control variables. These included: caregiver age, highest education attained, gender (i.e., 0 = male, 1 = female) race (i.e., African American, White, other or mixed race), and Hispanic/LatinX ethnicity (i.e., 0 = not Hispanic, 1 = Hispanic). 

*Family characteristics.* The number of children in the household was included as it has been found to be related to caregiver employment [38,39]. Having another adult with whom to share caregiving and income-earning responsibilities has also been found to shape caregiver employment decisions [38]. For that reason, we have included one question asking “Is there another adult in the household with whom you share caregiving responsibilities?” (i.e., 1 = no, 2 = yes). Household income was included as it is central to the conservation of resources theory (i.e., those with more resources are in a better position to protect from resource loss and to gain additional resources). Income was collected using 10 levels ranging from 1 = less than USD 5000 to 10 = USD 100,000 or more.

### 2.3. Analyses

We first conducted bivariate analyses to explore direct relationships between predictor and outcome variables. Chi-square tests were used to estimate relationships between categorical predictors and outcome variables. To compare means on continuous or interval predictor variables across groups with different employment outcomes, we used *t* tests.

To examine what factors contributed to employment outcomes, we applied binary logistic regression. Separate analyses were conducted for each of the employment outcome variables (i.e., gained employment, retained employment). Of primary interest was which, if any, services received by the child and family in the first six months after entering services were associated with employment outcomes at the six-month follow-up. We ran two sets of analyses. The first set tested whether receipt of any service was associated with the two caregiver employment outcomes. The base sample for these analyses was the full sample (*N* = 2455). However, sample sizes for each analysis varied according to employment status at baseline. Only caregivers unemployed at baseline were included in the analysis related to gaining employment (*N* = 1099). Similarly, only caregivers who were employed at baseline were included in the analysis predicting whether employment was retained (*N* = 1356).

The second set of logistic regression analyses examined what types of services were associated with employment outcomes. For these analyses, all six service types (described above) were included in each analysis. Only families that had received any service were included in these analyses (*N* = 2253), though sample sizes varied based on employment outcome as described above.

Because we were also interested in the child, caregiver, and family characteristics that may be associated with employment outcomes over time, we included covariates (assessed at baseline) that have been found to contribute to employment and service use including child internalizing and externalizing symptom severity, and the three caregiver strain variables. We also controlled for caregiver age, race, Hispanic ethnicity, gender, and family income at baseline. Age and income were entered as continuous variables, while gender and Hispanic ethnicity were entered as dichotomous variables. Two dummy variables were constructed to capture race. One compared African American caregivers to caregivers of all other races. The second compared White caregivers to caregivers of all other races. Being from another racial background (besides African American or White) or being of mixed race was the reference category.

For the binary logistic regression analyses, we applied a model-building approach. The initial model included only the service use variables. Model 2 added child symptom severity and caregiver strain. Model 3 added demographic variables including caregiver gender, age, education, race/ethnicity, the number of children in the home, and family income. 

## 3. Results

### 3.1. Bivariate Analyses

The results of bivariate analyses are summarized in Table 1 and described below by employment outcome.

*Gained employment*. Only caregivers who were not employed at baseline were included in these analyses (*N* = 1099). Of these, 178 were employed at the six-month follow-up. The group of caregivers who gained employment had statistically significantly lower mean age, higher educational attainment, and had more children living in the home. White caregivers were more likely to gain employment than caregivers in other race categories.

*Retained employment*. Of the 1356 caregivers who were employed at baseline, 172 reported not having been employed in the six months prior to follow-up data collection. Caregivers who lost employment had younger children, lower educational attainment, more children in the household, and lower household income at baseline. They also reported greater objective strain, on average.

### 3.2. Regression Analyses

The first set of analyses reported in Table 2 examined whether receiving any service was associated with employment outcomes. Across the entire sample, 8.3% of families reported receiving no services in the six months between baseline and follow-up (i.e., the services they received occurred in the 30-day window before baseline data collection).

The second set of analyses seen in Table 3 focused on the impact that different types of services had on employment. Of those who received a service, 10.4% used behavioral aides, 7% used day treatment, 49.3% used medication services, 15.3% used residential care, 31.3% used formal family services, and 65.6% used family support services.

For both employment outcomes, Model 3 (including all predictor variables) was the best-fitting model. In Table 2 and Table 3, we also present findings for Model 1 in order to show the direct relationships between service use variables and employment outcome before the introduction of other variables. We report odds ratios (ORs) and 95% confidence intervals (CIs) as estimates of the likelihood of experiencing the given employment outcome. ORs greater than 1 indicate a positive relationship; those less than 1 indicate a negative relationship. For each employment outcome, we first describe results for analyses examining the receipt of any service, followed by a description of results for analyses examining types of services.

*Gained employment.* Only caregivers who reported no employment at baseline were included in these analyses. In Model 1, receipt of any service significantly predicted the likelihood of gaining employment (*χ*^2^ = 5.64 (1), *p* < 0.05) (Table 2). Having received any service was positively associated with unemployed caregivers acquiring employment in the six months after baseline (*OR* = 1.89, *p* < 0.05). Model 2 was not statistically significant (*χ*^2^ = 9.47 (6), *p* = 0.15). Adding all covariates in Model 3 significantly improved model fit over Model 1 (Δ*χ*^2^ = 56.28 (Δ*df* = 15), *p* < 0.001). Use of any service remained a significant predictor when covariates were added to the analysis (*OR* = 1.87, *p* < 0.05), indicating that caregivers whose child and/or family received any mental health or support service were more likely to gain employment than caregivers of children/families who did not receive a service. In addition, being an older caregiver reduced the likelihood of gaining employment (*OR* = 0.95, *p* < 0.001). Having more education (*OR* = 1.15, *p* < 0.001) was related to gaining employment. Being African American reduced the likelihood of gaining employment (*OR* = 0.50, *p* < 0.05).

In analyses examining what types of services are related to gaining employment (see Table 3), use of behavioral aide services and medication services were directly related to gaining employment in Model 1 (*χ*^2^ = 12.25 (6), *p* = 0.057). Model 2 was not significant (*χ*^2^ = 17.05 (11), *p* = 0.11). Model 3 significantly improved model fit over Model 1 (Δ*χ*^2^ = 51.65 (Δ*df* = 15), *p* < 0.001). In Model 3, receiving behavioral aide services significantly increased the likelihood of gaining employment (*OR* = 2.15, *p* < 0.05). However, use of medication services was no longer a significant predictor when other covariates were added to the model. Younger caregiver age (*OR* = 0.95, *p* < 0.001) and higher educational attainment (*OR* = 1.14, *p* < 0.01) significantly increased the likelihood of gaining employment, while being African American reduced the likelihood (*OR* = 0.39, *p* < 0.05).

*Retained employment.* These analyses included only caregivers who reported having been employed at baseline. In the first set of analyses that tested use of any service (see Table 2), neither Model 1 (*χ*^2^ = 0.07 (1), *p* = 0.79) nor Model 2 (*χ*^2^ = 10.96 (6), *p* = 0.09) significantly predicted employment retainment. However, Model 3 explained a significant proportion of the variance in the outcome variable (*χ*^2^ = 66.24 (16), *p* < 0.001). Use of any service was not related to retaining employment. Experiencing higher objective strain reduced the likelihood of retaining employment (*OR* = 0.61, *p* < 0.001). In other words, caregivers who reported more strain at baseline were more likely to lose employment in the subsequent six months. In addition, caregivers with higher household incomes at baseline were more likely to retain employment in the subsequent 6 months (*OR* = 1.25, *p* < 0.001) (i.e., less likely to lose employment). Caregivers caring for an older child were also more likely to retain employment (*OR* = 1.06, *p* < 0.05).

In Model 1 of the second set of analyses examining service type, caregivers whose children used residential services were more likely to retain employment (*OR* = 1.64 *p* < 0.05). However, Model 1 did not predict a significant proportion of the variance in the employment retainment variable (*χ*^2^ = 11.76 (6), *p* = 0.067). While Model 2 was significant (*χ*^2^ = 24.22 (11), *p* < 0.05), Model 3 provided superior fit over Model 2 (Δ*χ*^2^ = 50.19 (Δ*df* = 10), *p* < 0.001). In Model 3, residential services remained a significant predictor after other variables were included in the analysis (*OR* = 1.58, *p* < 0.05). None of the other types of services significantly predicted loss of employment. Greater objective strain at baseline (*OR* = 0.62, *p* < 0.001) reduced the likelihood of retaining employment (i.e., increased the risk of losing employment). Older child age (*OR* = 1.06, *p* < 0.05) and higher family income at baseline (*OR* = 1.26, *p* < 0.001) increased the likelihood of retaining employment.

## 4. Discussion

The conservation of resources theory describes cyclical resource loss and gain over time and its role as a contributor to stress. Caring for a child with disabling emotional and behavioral challenges can threaten a family’s resources through the cost of specialized services, reduction in informal support systems, and time required to participate in services [40,41]. In many families, caregiver employment is central to efforts to acquire and maintain resources. However, caring for a child with emotional and behavioral disorders can negatively impact caregiver employment [42]. Observers have noted that supportive services can improve work–life integration for caregivers of children with emotional and behavioral disorders [43].

In this study, we used the conservation of resources theory to guide the examination of employment outcomes, over a 12-month period, among caregivers of children with emotional and behavioral disorders. Findings from this study are consistent with the conservation of resources theory.

### 4.1. Employment and Service Use

Services are critical resources for families caring for children with emotional and behavioral disorders. It is consistent with the conservation of resources theory that receipt of services would help families gain more resources and protect resources they already have. These findings provide some support for that theory.

Our study revealed that accessing services can improve some employment outcomes. It is notable that the services in this array were not specifically designed for the purpose of supporting caregiver employment. In fact, the pursuit of mental health treatment (e.g., service planning, treatment sessions, behavior management training) can create additional demands that may interrupt a caregiver’s workday. The families in this study were receiving services in federally funded systems of care. A fundamental value of systems of care is that services should be family driven. It may be, therefore, that these service systems attended to caregivers’ self-identified needs, and that may have included efforts to support participation in the paid labor force. However, this cannot be determined with these data.

The relationship between service/supports and caregiver employment differed depending on initial employment status, as well as type of services received. For caregivers who were unemployed at baseline, accessing any service for their child was associated with gaining employment. It may be that receipt of services reduced symptoms and related caregiving demands (e.g., need to pick child up at school because of disruptive behavior), allowing the caregiver to pursue employment. Although use of any service was not associated with retaining employment, specific service types were related to employment among families who received any services.

For families who accessed any services, receiving behavioral aide services appears to have helped unemployed caregivers gain employment. Behavioral aides often work in schools. Perhaps having a behavioral aide in the child’s classroom helps children remain in the classroom throughout the school day, making it possible for caregivers to pursue employment because they anticipate fewer work disruptions [44]. This explanation is consistent with previous research demonstrating that children with more severe symptoms miss more days of school, which was associated with caregivers not being employed at a given point in time [10].

The finding that employed caregivers of children who used residential services were less likely to lose employment is intriguing. The residential services considered in this study ranged from psychiatric hospitalization with relatively short stays, to longer term use of residential treatment facilities. In any case, residential services are used as a last resort when management at home is too challenging. It is possible that having the child out of the home during times of acute worsening of symptoms and functioning allows caregivers to work with less interruption.

Use of family support services was not associated with either employment outcome. Considering the types of family support services caregivers reported receiving (i.e., respite, flexible funds for non-traditional support, transportation to services, peer-to-peer support), it is likely that these types of supports were not provided specifically to allow caregivers to work. The finding that receipt of day treatment services was not associated with employment outcomes was unexpected. One might have expected day treatment services to reduce work disruptions during traditional work hours because school staff would have the specialized skills to respond to challenging child behavior without requiring caregivers to leave work. However, the data did not support this expectation. 

### 4.2. Employment and Child, Caregiver, and Family Characteristics

Beyond services, several child characteristics were associated with caregiver employment outcomes. Severity of child internalizing and externalizing problems at baseline was not found to be related to employment outcomes in regression analyses. This is consistent with a previous study that found no relationship between severity of child symptom severity and work engagement among working caregivers when caregiver strain and other covariates were included in the model [22]. Although that study found that child internalizing and externalizing symptom severity had direct bivariate relationships with absenteeism from work, caregiver strain was found to mediate the relationship between child symptom severity and missed work.

Several caregiver and family characteristics had statistically significant relationships with employment outcomes. In both sets of regression analyses, caregivers who reported more objective strain at baseline were more likely to lose their job in the subsequent six months, compared to caregivers who reported less objective strain, with all other variables remaining equal. These findings are consistent with a previous study of working caregivers that found that greater objective caregiver strain was associated with missing more days of work in the six months before their child entered services [22]. These finding suggest that the observable onerous events (e.g., caregiver having difficulty attending to responsibilities, child getting in trouble with neighbors or in school) compromise caregivers’ ability to remain in the paid labor force.

Neither of the subjective strain dimensions predicted employment outcomes when regression analyses controlled for other variables. This finding differs from previous research that subjective externalized strain (i.e., feelings of anger, resentment, embarrassment) was associated with greater workforce engagement including working more months in the six months before bringing their children to services, working more hours per week, and missing fewer days of work [22]. The previous study speculated that caregivers who reported feeling more subjective externalized strain (e.g., anger, embarrassment, resentment) may have reached the point that they would not allow the youth’s challenges to disrupt their work lives [22]. It is also possible that having to juggle work and extraordinary caregiving demands leads caregivers to feel more angry and resentful. That study, however, included only caregivers who had experienced some employment in the six months prior to their child entering services, and did not examine employment outcomes after that point. Taken together, these studies suggest that while subjective externalized strain may be associated with extent of workforce engagement for working caregivers at a given point in time, it does not appear to explain changes in employment over time. 

Study findings related to income and education support the conservation of resources theory. Lower household income at baseline was associated with poorer employment outcomes, potentially exacerbating the cycle of resource loss. For example, caregivers who retained employment between baseline and six-month follow-up reported having higher family incomes at baseline, on average. It stands to reason that families with higher incomes are better able to use their resources to buffer caregivers from employment loss (e.g., specialized child care that reduces work disruptions). It is also possible that caregivers with higher incomes are in jobs that allow more personal days off or have flexible schedules that allow them to respond to disruptions (e.g., able to make up lost work in the evening when they need to leave work early to pick up a child from day care or school). Consistent with employment in the general population, caregivers in this study who had higher educational attainment were more likely to gain employment. Taken together, these findings suggest that families with more resources are better able to protect themselves against further resource loss.

Other demographic variables were related to employment in expected ways. Being older reduced the likelihood of gaining employment. This may be due, in part, to the number of grandparents who are serving as caregivers in this sample and may be retired. African American caregivers, too, were less likely to gain employment. These relationships have been well documented for the general public, as well.

### 4.3. Limitations

Several cautions in the interpretation of findings are warranted. Related to service use, it is not possible to determine causal directionality. Findings indicate that receipt of services can help caregivers improve employment outcomes. However, it is also possible that children with working caregivers are more likely to receive services because their caregivers can afford them or because they are more likely to have health insurance and other benefits (e.g., paid sick leave).

Another concern is that caregivers served as informants for all variables. This is a particular concern regarding the service use variables. Previous studies have found that caregivers do not always report their children’s service use with accuracy and precision. Caregiver reports of the number of sessions/service contacts are especially problematic. In this study, we prudently relied on caregivers to only report whether a type of service was received, and not the number of contacts. Nonetheless, future studies should utilize independent sources of information on service use (e.g., service claims data, administrative data). This will also allow investigation into whether quantity and intensity of services are related to employment outcomes.

In addition, the employment variables relied on a single item about employment over the previous six months. This question was asked at baseline and at 6-month follow-up. Future studies should collect more detailed employment information including nature of the work (e.g., part-time versus full-time), changes in jobs and roles (e.g., moving to another position to accommodate child needs), and extent of employer support (e.g., flexibility of work schedule, amount of personal time off). 

Two cautions should be noted related to the sample. First, this was a convenience sample. No claims can be made that this sample is representative of all children receiving mental health services. Second, the loss of 25.4% of the sample due to missing data is concerning. No differences between included and excluded families were found for caregiver education, race/ethnicity, number of children in the household, or family income. However, compared to the original sample, the caregivers whose data were used for this study were older, more likely to be women, and reported that their children had more severe internalizing and externalizing symptoms. Included caregivers also reported more caregiver strain. These considerations should be taken into account when considering to whom these findings may generalize.

There were also secular changes in the broader society that may have impacted these findings. Specifically, the worldwide recession of 2007–2008 occurred during the study’s observation period. To ascertain the extent of the recession on these employment outcomes, we conducted post hoc analyses. In 2 × 3 contingency tables, we cross-tabulated employment outcomes (e.g., gained employment, retained employment) with whether the family entered the study before the recession (i.e., 2004–2006), during the recession (i.e., 2007 or 2008), or after the recession (i.e., 2009–2011). We found that caregivers who entered the study after the recession were less likely to gain employment in the subsequent 6 months compared to those who entered before and during the recession (*Χ*^2^ = 6.44 (2), *p* < 0.04). However, that relationship disappeared when service variables were entered into Model 1 of the regression analyses. This suggests that entering the study during the recession had a direct bivariate relationship with gaining employment, but it provided no unique explanation of variance in employment, for this sample. Year of entry into the study was not found to be associated with whether caregivers retained employment in cross-tabulation or regression analyses.

## 5. Conclusions

Despite these limitations, findings from this study reveal the need to support families as they care for their children with emotional and behavioral challenges. Taking a conservation of resources perspective, we argue that behavioral health services and other family supports may increase caregiver participation in the paid labor force, allowing for the accumulation and protection of resources. Additional research is needed to assess the impact of specialized services and supports for youth with mental health challenges on caregiver employment [45].

Service providers working with families should consider findings from this study. As the field continues to move toward meaningful family-driven care, practitioners are called upon to help strengthen families’ ability to support their children with emotional and behavioral disorders. These findings suggest that improving caregiver employment outcomes may be an appropriate goal for many families. Service providers should also work to reduce work disruptions for caregivers as they strive to promote family involvement in treatment. Explicit efforts to support caregiver employment may mitigate the risk of families experiencing downward cycles of resource loss. 

## Figures and Tables

**Table 1:** Descriptive Statistics by Work Outcome for Caregivers of Children and Youth in Systems of Care.

	Employment Outcomes			
	Gained Employment		Retained Employment	
	No	Yes	No	Yes
Characteristics at baseline	*N* = 921	*N* = 178	*N* = 172	*N* = 1184
	*M* (*SD*)	*M* (*SD*)	*M* (*SD*)	*M* (*SD*)
Child Variables				
Internalizing	66.03 (10.06)	65.16 (9.71)	65.31 (9.98)	65.11 (9.67)
Externalizing	69.96 (9.37)	69.79 (9.74)	70.36 (9.45)	70.21 (8.98)
Age	11.82 (3.17)	11.45 (3.20)	11.23 (3.57)	11.86 (3.23) *
Caregiver Variables				
Objective strain	2.65 (1.05)	2.66 (1.08)	2.94 (1.06) *	2.80 (1.03)
Subj. ext. strain	2.33 (0.96)	2.39 (0.99)	2.39 (1.02)	2.49 (0.97)
Subj. int. strain	3.59 (0.98)	3.66 (1.02)	3.66 (0.96)	3.69 (0.96)
Age	41.94 (11.40) ***	37.49 (9.91)	38.65 (9.76)	39.39 (8.60)
Education	11.65 (2.47) *	12.10 (2.10)	12.50 (2.14) *	12.85 (2.05)
Family Variables				
Number of children	2.67 (1.55) *	2.96 (1.59)	2.72 (1.77) *	2.45 (1.35) *
Income	3.42 (2.18)	3.21 (2.29)	3.99 (2.42) ***	5.23 (2.36)
	*N* (%)	*N* (%)	*N* (%)	*N* (%)
Other caregiver in home	687 (74.6)	137 (77)	134 (77.9)	937 (79.1)
Caregiver Variables				
Female	873 (94.8)	168 (94.4)	158 (91.9)	1089 (92)
Race/Ethnicity				
African Amer.	251 (27.4)	66 (37.1)	58 (33.7)	336 (28.4)
White	604 (65.6) *	101 (56.7)	101 (58.7)	783 (66.1)
Other	66 (7.2)	11 (6.2)	13 (7.6)	65 (5.5)
Hispanic	194 (21.1)	39 (21.9)	29 (16.6)	158 (13.3)

Note. * *p* < 0.05, *** *p* < 0.001.

**Table 2:** Regression Analyses Predicting Employment Outcome: Any Use of Services.

	Employment Outcomes			
Characteristics at Baseline	Gained Employment *N* = 1099		Retained Employment *N* = 1356	
**Model 1 Fit Statistics**	*Χ*^2^ = 5.64 (1) *p* < 0.05		*Χ*^2^ = 0.07 (1) *p* = 0.79	
	*OR*	*CI*	*OR*	*CI*
Use of any service	1.89 *	1.14–3.13	0.92	0.52–1.63
**Model 3 Fit Statistics**	*Χ*^2^ = 61.92 (16) *p* < 0.0001		*Χ*^2^ = 66.24 (16) *p* < 0.0001	
	*OR*	*CI*	*OR*	*CI*
Use of any service	1.87 *	1.09–3.22	0.79	0.43–1.44
Child characteristics				
Internalizing symptoms	0.99	0.97–1.0	1.00	0.97–1.02
Externalizing symptoms	0.99	0.97–1.02	1.02	0.99–1.04
Age	0.99	0.94–1.05	1.06 *	1.00–1.12
Caregiver characteristics				
Objective strain	0.93	0.73–1.19	0.61 ***	0.47–0.78
Subjective ext. strain	1.10	0.89–1.36	1.16	0.93–1.45
Subjective int. strain	1.20	0.93–1.54	1.23	0.95–1.60
Male	1.81	0.86–3.85	0.91	0.48–1.70
Age	0.95 ***	0.93–0.97	0.99	0.97–1.01
Education	1.15 ***	1.05–1.26	1.01	0.93–1.10
African American	0.50 *	0.23–0.98	1.01	0.50–2.01
White	0.92	0.46–1.87	0.81	0.41–1.60
Hispanic	0.69	0.44–1.10	1.33	0.82–2.15
Family Characteristics				
Number of children in household	1.05	0.94–1.17	0.90	0.81–1.01
Other caregiver in home	1.21	0.82–1.80	1.10	0.74–1.65
Household income	0.99	0.91–1.08	1.25 ***	1.15–1.35

Note. * *p* < 0.05, *** *p* < 0.001.

**Table 3:** Regression Analyses Predicting Employment Outcome: Types of Services.

	Employment Outcomes			
Characteristics at Baseline	Gained Employment *N* = 1008		Retained Employment *N* = 1245	
**Model 1 Fit Statistics**	*Χ*^2^ = 12.25 (6) *p* = 0.057		*Χ*^2^ = 11.76 (6) *p* = 0.067	
	*OR*	*CI*	*OR*	*CI*
Day treatment	1.09	0.52–2.28	1.30	0.69–2.46
Behavioral aide	2.09 *	1.03–4.27	0.93	0.53–1.61
Medication services	1.47 *	1.03–2.11	0.93	0.66–1.31
Residential services	0.68	0.43–1.09	1.64 *	1.06–2.53
Family support services	0.91	0.63–1.31	1.41 ^†^	0.67–2.06
Formal family services	1.14	0.78–1.67	0.68 ^†^	0.46–1.02
**Model 3 Fit Statistics**	*Χ*^2^ = 63.90 (21) *p* < 0.0001		*Χ*^2^ = 74.41 (21) *p* < 0.0001	
	*OR*	*CI*	*OR*	*CI*
Service use variables				
Day treatment	1.25	0.58–2.70	1.28	0.66–2.51
Behavioral aide	2.15 *	1.03–4.50	0.87	0.49–1.53
Medication services	1.44 ^†^	0.97–2.14	0.95	0.65–1.40
Residential services	0.67	0.41–1.09	1.58 *	0.99–2.52
Family support services	1.06	0.72–1.56	1.30	0.88–1.63
Formal family services	1.12	0.75–1.66	0.71	0.47–1.07
Child characteristics				
Internalizing symptoms	0.99	0.97–1.01	1.00	0.98–1.02
Externalizing symptoms	1.00	0.97–1.03	1.01	0.99–1.04
Age	0.97	0.91–1.03	1.06 *	1.00–1.13
Caregiver characteristics				
Objective strain	0.97	0.75–1.26	0.62 ***	0.47–0.81
Subjective ext. strain	1.04	0.83–1.31	1.21	0.96–1.54
Subjective int. strain	1.21	0.92–1.58	1.25	0.95–1.66
Male	1.28	0.54–3.04	0.93	0.47–1.83
Age	0.95 ***	0.93–0.97	0.99	0.97–1.01
Education	1.14 **	1.03–1.25	1.00	0.92–1.10
African American	0.39 *	0.17–0.89	0.83	0.40–1.72
White	0.67	0.30–1.51	0.75	0.37–1.51
Hispanic	0.69	0.42–1.13	1.47	0.88–2.46
Family Characteristics				
Number of children	1.05	0.93–1.17	0.92	0.82–1.04
Other caregiver in home	1.29	0.84–1.97	1.17	0.76–1.78
Household income	0.98	0.90–1.08	1.26 ***	1.16–1.37

^†^ *p* < 0.07, * *p* < 0.05, ** *p* < 0.01, *** *p* < 0.001.

## Data Availability

Restrictions apply to the availability of these data. Data were obtained through the Substance Abuse and Mental Health Services Administration. Requests for data should be directed to their Substance Abuse and Mental Health Data Archive (https://www.datafiles.samhsa.gov/about-us).
